# VASOMOTOR SYMPTOMS AND DIET QUALITY

**DOI:** 10.1093/geroni/igad104.2452

**Published:** 2023-12-21

**Authors:** Pilar Thangwaritorn, Jaime Perales-Puchalt, Melanie Meister, Tiffany Schwasinger-Schmidt, Sandra Billinger, Briana Bright, Amber Watts

**Affiliations:** University of Kansas, Lawrence, Kansas, United States; University of Kansas Alzheimer’s Disease Research Center, Fairway, Kansas, United States; University of Kansas School of Medicine, Kansas City, Kansas, United States; University of Kansas School of Medicine-Wichita, Wichita, Kansas, United States; University of Kansas Medical Center, Kansas City, Kansas, United States; Kansas City, Kansas, United States; University of Kansas, Lawrence, Kansas, United States

## Abstract

During the menopausal transition, women report a variety of physical changes including hot flashes and nights sweats, known as vasomotor symptoms, which are associated with later life health outcomes including vascular disease and Alzheimer’s risk. Previous studies suggest that maintaining a healthy diet may alleviate vasomotor symptoms, however, few studies have investigated this association. We examined the potential association between diet quality and vasomotor symptoms in women ages 40-60 years. Recruitment is ongoing and currently consists of N = 44 women (Mage = 50.59, SD = 5.96) from the Kansas City and Wichita areas. We assessed vasomotor symptoms using two questions from the Greene Climacteric Scale and measured diet quality with the Rapid Eating Assessment for Participants-shortened version. We evaluated the association between diet quality and vasomotor symptoms using linear regression. Overall, women in our sample reported moderate diet quality and were moderately bothered by hot flashes and night sweats. The association of diet and vasomotor symptoms was not statistically significant accounting for covariates, possibly related to sample size (β = 6.024, p = .307). However, lower education levels are associated with a greater degree of vasomotor symptoms (β = -0.876, p = .011). Future research could investigate specific types of foods (e.g., vegetables, proteins, etc.) that influence vasomotor symptoms and the associated health outcomes in women who are post-menopausal, including vascular disease and cognitive decline. Lifestyle factors such as diet may be an intervention strategy to reduce disease that emerges during the menopausal transition.

